# Multi-Modal Imaging of Neural Correlates of Motor Speed Performance in the Trail Making Test

**DOI:** 10.3389/fneur.2015.00219

**Published:** 2015-10-27

**Authors:** Julia A. Camilleri, Andrew T. Reid, Veronika I. Müller, Christian Grefkes, Katrin Amunts, Simon B. Eickhoff

**Affiliations:** ^1^Research Centre Jülich, Institute of Neuroscience and Medicine (INM-1), Jülich, Germany; ^2^Institute of Clinical Neuroscience and Medical Psychology, Heinrich Heine University, Düsseldorf, Germany; ^3^Department of Neurology, University Hospital Cologne, Cologne, Germany; ^4^C. and O. Vogt Institute for Brain Research, Heinrich Heine University, Düsseldorf, Germany

**Keywords:** trail-making test, motor speed, inferior frontal sulcus, voxel-based morphometry, resting state fMRI, meta-analytic connectivity modeling, structural covariance, probabilistic tractography

## Abstract

The assessment of motor and executive functions following stroke or traumatic brain injury is a key aspect of impairment evaluation and used to guide further therapy. In clinical routine, such assessments are largely dominated by pen-and-paper tests. While these provide standardized, reliable, and ecologically valid measures of the individual level of functioning, rather little is yet known about their neurobiological underpinnings. Therefore, the aim of this study was to investigate brain regions and their associated networks that are related to upper extremity motor function, as quantified by the motor speed subtest of the trail making test (TMT-MS). Whole-brain voxel-based morphometry and whole-brain tract-based spatial statistics were used to investigate the association between TMT-MS performance with gray-matter volume (GMV) and white-matter integrity, respectively. While results demonstrated no relationship to local white-matter properties, we found a significant correlation between TMT-MS performance and GMV of the lower bank of the inferior frontal sulcus, a region associated with cognitive processing, as indicated by assessing its functional profile by the BrainMap database. Using this finding as a seed region, we further examined and compared networks as reflected by resting state connectivity, meta-analytic connectivity modeling, structural covariance, and probabilistic tractography. While differences between the different approaches were observed, all approaches converged on a network comprising regions that overlap with the multiple-demand network. Our data therefore indicate that performance may primarily depend on executive function, thus suggesting that motor speed in a more naturalistic setting should be more associated with executive rather than primary motor function. Moreover, results showed that while there were differences between the approaches, a convergence indicated that common networks can be revealed across highly divergent methods.

## Introduction

Hand motor deficits are among the most common impairments following stroke (1). As a result, post-stroke assessment of motor functions is a key aspect of patient evaluation and is used to guide further therapy. In addition to fast but typically qualitative clinical assessments, this often involves neuropsychological tests of coordinated hand function. In practice, such assessments are still largely dominated by pen-and-paper tests. One example of such a simple pen-and-paper test is the motor speed subtest of the trail-making test (TMT-MS) from the Delis–Kaplan executive function system [D–KEFS; (2)]. This test measures the time that subjects take to manually trace a pre-specified trail. The TMT-MS requires the examinee to connect circles by following a dotted line, and aims to serve as a baseline measure of the motor component that should be shared by the other portions of the test. The results should thus provide information about the extent to which difficulty on the other TMT subtests probing higher, executive functions may be related to a motor deficit. However, the results of the TMT-MS cannot only be used as a baseline for other TMT subtests, but also provide information of drawing speed *per se*, and thus can be used by clinicians as an assessment of upper extremity motor function (2).

Pen-and-paper tests such as the TMT provide standardized and reliable valid measures of the individual level of functioning; however, rather little is yet known about their neurobiological underpinnings. Therefore, one aim of the current study is to investigate brain–behavior relationships with regard to upper extremity motor function, as quantified by the TMT-MS from the D–KEFS. Additionally, previous studies have demonstrated that while the brain can be subdivided into distinct modules based on functional and microstructural properties [reviewed in Ref. (3)], processes such as motor function are likely to involve the efficient integration of information across a number of such specialized regions. Due to this integrative nature of the brain, most higher mental functions are likely implemented as distributed networks (4), and it has therefore been suggested that an understanding of how a brain region subserves a specific task should require information regarding its interaction with other brain regions (3). Therefore, the current study additionally aims to investigate the networks associated with the regions we find to be related to TMT-MS performance.

A number of different approaches can be employed to investigate networks associated with a particular brain region. Task-free (seed-based) resting-state functional connectivity (RS-FC) refers to temporal correlations of a seed region with spatially distinct brain regions, when no task is presented (5, 6). Meta-analytic connectivity modeling (MACM) (7–9) investigates co-activation patterns between a seed region and the rest of the brain, by calculation of meta-analyses across many task-based fMRI experiments and paradigms stored in, e.g., the BrainMap database (10, 11). Structural covariance (SC) is based on the correlation patterns across a population of gray-matter characteristics such as volume or thickness (12, 13) that are thought to reflect shared mutational, genetic, and functional interaction effects of the regions involved (14, 15). While having conceptual differences, these three modalities all share the goal of delineating regions that interact functionally with a particular seed region. By contrast, probabilistic tractography (PT) focuses on white-matter anatomical connectivity obtained from diffusion-weighted images (DWI) by producing a measure of the likelihood that two regions are structurally connected (16, 17). Previous studies have reported convergence between RS and MACM (18–20), between RS and SC (21, 22), RS and fiber tracking (23–26), and between RS, MACM, and SC (27, 28). However, striking differences among the different connectivity approaches have also been found (26, 27).

In this study, we first used whole-brain voxel-based morphometry [VBM; (29)] and whole-brain tract-based spatial statistics [TBSS; (30)] to investigate the association between TMT-MS performance with gray-matter volume (GMV) and white-matter integrity, respectively. Using the result of these initial analyses as the seed region of interest, we further examined and systematically compared networks obtained through RS-fMRI, MACM, SC, and PT. The aim of these analyses was twofold. First, we sought to explore the relationship of brain morphology to a simple measure of hand motor function. Second, we aimed to characterize both the divergence and convergence of four unique approaches to quantifying brain connectivity.

## Materials and Methods

### Subjects

**Figure 1:**
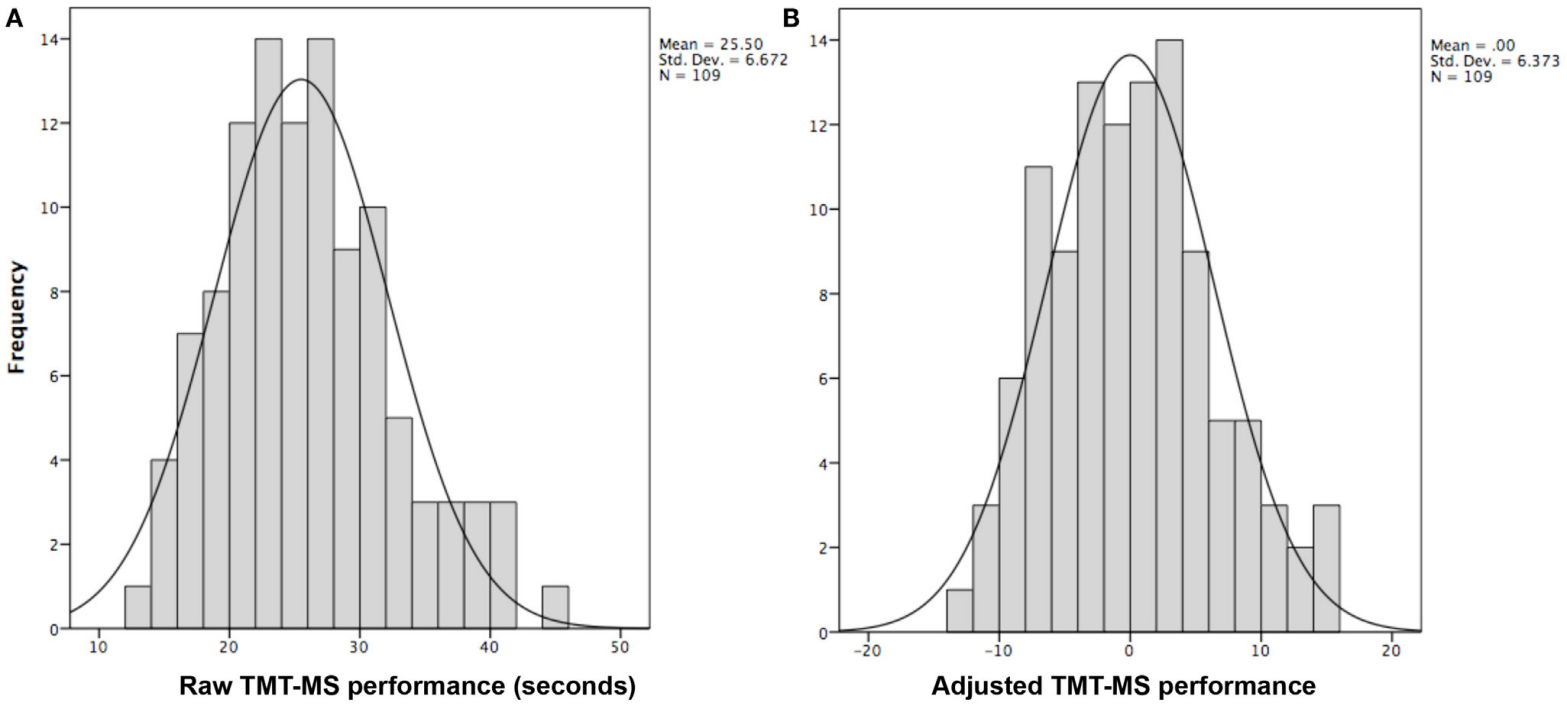
**Histograms showing distribution of TMT-MS performance**. **(A)** The distribution of the raw TMT-MS performance. **(B)** The distribution of the adjusted TMT-MS performance after effects of age, gender, handedness, and BDI scores were regressed out of the raw scores.

Data from the Enhanced Nathan Kline Institute – Rockland Sample^1^ (31) was used for all analyses except for meta-analytical connectivity modeling and functional characterization (where the BrainMap database was used). From this cohort, we used anatomical, RS, and DWI of subjects that had completed the TMT-MS, no current psychiatric diagnosis, a Beck depression inventory score (BDI) of less than 14 and did not exceed 3 SDs from the population mean. This resulted in a sample of 109 right-handed healthy volunteers between 18 and 75 years of age (mean age 40.39 ± 15.49; 37 males). First, effects of age, gender, handedness, and BDI score as known influences on hand motor speed (32, 33) were regressed out of the raw TMT-MS performance score (Figure 1A; Table 1). This resulted in an adjusted performance score, which indicated how much better or worse a subject performed than would be expected given these confounding factors (Figure 1B). The association of these adjusted scores with local GMV and white-matter integrity was then tested by carrying out whole-brain VBM and TBSS, respectively.

**Table 1:** **Characteristics of the cohort**.

Age	Gender	BDI	EHI
26	Male	4	80
20	Male	0	95
53	Male	0	55
48	Female	9	100
62	Female	5	90
18	Female	7	75
54	Female	0	95
18	Female	1	90
21	Male	4	85
62	Female	1	100
53	Male	3	75
22	Male	4	90
62	Female	12	100
54	Female	0	95
24	Female	1	85
44	Female	8	90
57	Female	2	95
44	Female	3	70
51	Male	7	70
63	Female	0	80
26	Female	1	60
59	Male	4	95
30	Male	0	85
50	Female	1	90
26	Female	2	75
18	Male	0	80
24	Female	10	95
64	Female	0	95
47	Male	4	100
38	Female	0	80
23	Female	1	70
42	Female	8	85
59	Female	2	100
26	Male	5	100
18	Male	3	90
19	Male	1	100
27	Female	12	60
20	Female	3	100
56	Female	5	100
18	Male	4	85
30	Male	4	55
58	Female	6	95
52	Female	3	85
38	Male	1	65
64	Male	5	80
41	Female	2	100
49	Female	5	60
57	Female	8	60
40	Female	3	80
48	Female	0	100
36	Female	1	100
20	Male	5	90
60	Female	3	75
59	Male	2	85
52	Female	8	100
41	Male	1	70
26	Female	7	75
51	Female	5	75
61	Female	0	80
58	Male	5	80
56	Female	0	65
54	Female	4	95
27	Male	5	60
42	Female	9	70
31	Female	7	100
21	Female	1	100
18	Male	3	90
48	Female	3	85
20	Female	5	55
60	Female	1	100
20	Female	1	90
50	Female	2	90
62	Male	7	70
18	Male	2	85
57	Female	1	100
24	Female	0	95
26	Female	0	80
57	Female	5	85
19	Male	2	70
49	Male	0	60
23	Female	2	85
58	Female	5	55
55	Male	4	80
41	Female	5	100
41	Female	0	100
25	Female	2	75
49	Female	0	90
49	Female	1	100
21	Female	6	75
50	Male	1	85
19	Male	3	65
59	Male	3	85
41	Male	0	80
44	Male	13	100
20	Female	13	85
47	Male	5	90
21	Male	2	55
47	Female	7	55
55	Female	1	90
23	Female	13	100
61	Male	1	80
52	Female	0	100
20	Male	10	60
51	Female	0	65
42	Female	0	100
21	Female	0	80
36	Female	8	100
43	Female	9	85
43	Female	5	95

### Delis–Kaplan Executive Function System: Trail-Making Test – Motor Speed

The Delis–Kaplan executive function system: trail-making test (D–KEFS TMT) consists of five different conditions (2). For the current study, we were exclusively interested in the TMT-MS, which requires participants to trace over a dotted line as quickly as possible while making sure that the line drawn touches every circle along the path. In particular, the participant is prompted to focus on speed rather than neatness but has to make sure that the line touches every circle along the path. If the line departs from the dotted line or is not correctly connected to the next circle, the participant is stopped immediately and redirected to the dotted line while keeping the stopwatch running. The scoring measure is the time (in seconds) that the participant needs to complete the task.

### Relationship Between TMT-MS Performance and Gray-Matter Volume

#### Whole-Brain VBM Analysis

The association between regional GMV and individual performance (adjusted for the potentially confounding effects of age, gender, handedness, and BDI), was investigated by performing a whole-brain VBM analysis. This analysis used the anatomical T1-weighted images of the 109 subjects described above. These scans were acquired in sagittal orientation on a Siemens TimTrio 3T scanner using an MP-RAGE sequence (TR = 1900 ms, TE = 2.52 ms, TI = 900 ms, flip angle = 9°, FOV = 250 mm, 176 slices, voxel size = 1 mm × 1 mm × 1 mm). Images were preprocessed using the VBM8 toolbox in SPM8 using standard settings, namely spatial normalization to register the individual images to ICBM-152 template space, and segmentation, wherein the different tissue types within the images are classified. The resulting normalized gray-matter segments, modulated only for the non-linear components of the deformations into standard space, were then smoothed using an 8 mm isotropic full-width-half-maximum (FWHM) kernel, and finally assessed for significant correlation between GMV and the adjusted TMT-MS performance scores. Age, gender, BDI scores, and Edinburgh handedness inventory (EHI) scores were used as covariates together with the adjusted TMT-MS performance scores, leading to an analysis of partial correlations between GMV and TMT-MS. As we modulated the gray-matter probability maps by the non-linear components only to represent the absolute amount of tissue corrected for individual brain size, we did not include total brain volume as an additional covariate in the analysis. That is, given that the correction for inter-individual differences in brain volume was applied directly to the data it was not performed (a second time) as part of the statistical model. Statistical significance using non-parametric permutation inference was assessed at *p* < 0.05 [family-wise error (FWE) corrected for multiple comparisons].

#### Whole-Brain TBSS Analysis

A TBSS whole-brain analysis was performed to investigate the association between white-matter volume and adjusted TMT-MS performance. DWI from the same group of 109 volunteers acquired on a 3T TimTrio Siemens scanner (137 directions, *b* = 1,500 s/mm^2^) were used. Preprocessing was performed according to standard protocols using FSL^2^. The DWI data were first corrected for head-motion and eddy-current effects of the diffusion gradients. The b0 images were averaged and skull-stripped using BET (34) to create the analysis mask. Within this mask, a simple diffusion-tensor model was estimated for each voxel. Finally, non-linear deformation fields between the diffusion space and the ICBM-152 reference space were computed using FSL’s linear (FLIRT) (35, 36), and non-linear (FNIRT) image registration tools (37). These allow mapping between the individual (native) diffusion space and the ICBM-152 reference space; i.e., the same space to which also the VBM and RS (as described below) data are also registered. The FA images were hereby normalized into standard space and then merged to produce a mean FA image. This was in turn used to generate a skeleton representing all fiber tracts common to all subjects included in the study (30, 38). The maximal FA scores of each individual FA image were then projected onto the mean FA skeleton. This projection aims to resolve any residual alignment problems after the initial non-linear registration (38). The resulting skeleton was then used to perform a multi-covariate analysis, using age, gender, BDI scores, EHI scores, and TMT-MS scores. Statistical significance using non-parametric permutation inference was again assessed at *p* < 0.05 multiple comparisons.

#### Seed Definition and Functional Characterization

The regions revealed by the initial VBM analysis were functionally characterized based on the behavioral domain meta-data from the BrainMap database^3^ (10, 11, 39), using both forward and reverse inference, as performed in previous studies (40, 41). Behavioral domains, which have been grouped for the purpose of the database, describe the cognitive processes probed by an experiment. Forward inference is the probability of observing activity in a brain region, given knowledge of the psychological process; whereas reverse inference is the probability of a psychological process being present, given knowledge of activation in a particular brain region. The results of both the forward and reverse inferences will be defined by the number and frequency of tasks in the database. In the forward inference approach, the functional profile was determined by identifying taxonomic labels for which the probability of finding activation in the respective region/set of regions was significantly higher than the overall (*a priori*) chance across the entire database. That is, we tested whether the conditional probability of activation given a particular label [P(Activation|Task)] was higher than the baseline probability of activating the region(s) in question *per se* [P(Activation)]. Significance was established using a binomial test [*p* < 0.05, corrected for multiple comparisons using false discovery rate (FDR)]. In the reverse inference approach, the functional profile was determined by identifying the most likely behavioral domains, given activation in a particular region/set of regions. This likelihood P(Task|Activation) can be derived from P(Activation|Task) as well as P(Task) and P(Activation) using Bayes’ rule. Significance (at *p* < 0.05, corrected for multiple comparisons using FDR) was then assessed by means of a chi-squared test.

### Multi-Modal Connectivity Analyses

Multi-modal connectivity analyses were used to further characterize the results from the initial VBM analysis. In particular, we investigated; (1) RS-FC, inferred through correlations in the blood–oxygen-level-dependent (BOLD) signal obtained during a task-free, endogenously controlled state (5, 6); (2) MACM, revealing co-activation during the performance of external task demands (7, 8); (3) SC, identifying long-term coordination of brain morphology (15); and (4) probabilistic fiber tracking, providing information about anatomical connectivity by measuring the anisotropic diffusion of water in white-matter tracts (16, 17).

All the analyses were approved by the local ethics committee of the Heinrich Heine University Düsseldorf.

#### Task-Independent Functional Connectivity: Resting-State

A seed-based RS analysis was used to investigate the task-independent FC of the seed region (5, 6). RS-fMRI images of the 109 subjects described above were used. During the RS acquisition, subjects were instructed to not think about anything in particular but not to fall asleep. Images were acquired on a Siemens TimTrio 3T scanner using BOLD contrast [gradient-echo EPI pulse sequence, TR = 1.4 s, TE = 30 ms, flip angle = 65°, voxel size = 2.0 mm × 2.0 mm × 2.0 mm, 64 slices (2.00 mm thickness)].

Data were processed using SPM8 (Wellcome Trust Centre for Neuroimaging, London^4^). The first four scans were excluded prior to further analyses and the remaining EPI images were then corrected for head movement by affine registration which involved the alignment to the initial volumes and then to the mean of all volumes. No slice time correction was applied. The mean EPI image for each subject was then spatially normalized to the ICBM-152 reference space by using the “unified segmentation” approach. (42). The resulting deformation was then applied to the individual EPI volumes. Furthermore, the images were smoothed with a 5-mm FWHM Gaussian kernel so as to improve the signal-to-noise ratio and to compensate for residual anatomic variations. The time-series of each voxel were processed as follows: spurious correlations were reduced by excluding variance that could be explained by the following nuisance variables: (i) the six motion parameters derived from the re-alignment of the image; (ii) their first derivatives; (iii) mean gray matter, white matter, and CSF signal. All nuisance variables entered the model as both first- and second-order terms. The data were then band-pass filtered preserving frequencies between 0.01 and 0.08 Hz. The time-course of the seed was extracted for every subject by computing the first eigenvariate of the time-series of all voxel within the seed. This seed time-course was then correlated with the time-series of all the other gray-matter voxels in the brain using linear (Pearson) correlation. The resulting correlation coefficients were transformed into Fisher’s *z*-scores and tested for consistency across subjects by using a second-level ANOVA including age, gender, BDI scores, and EHI scores as covariates of no interest. Results were corrected for multiple comparisons using threshold-free cluster enhancement, a method that has been suggested to improve sensitivity and provide more interpretable output than cluster-based thresholding [TFCE; (43)], and FWE-correction at *p* < 0.05.

#### Task-Dependent Functional Connectivity: Meta-Analytic Connectivity Modeling

The whole-brain connectivity of the seed was characterized using a task-dependent approach by carrying out MACM. This method looks at FC as defined by task activation from previous fMRI studies and benefits from the fact that a large number of such studies are normally presented in a highly standardized format and stored in large-scale databases (9). Thus, MACM is based on the assessment of brain-wise co-activation patterns of a seed region across a large number of neuroimaging experiment results (7). All experiments that activate the particular seed region are first identified and then used in a quantitative meta-analysis to test for any convergence across all the activation foci reported in these experiments (9). Any significant convergence of reported foci in other brain regions as the seed was considered to indicate consistent co-activation with the seed. For this study, we used the BrainMap database to identify studies reporting neural activation within our seed region^5^ (10). A coordinate based meta-analysis was then used to identify consistent co-activations across the experiments identified by using activation likelihood estimation (ALE) (44–46). This algorithm treats the activation foci reported in the experiments as spatial probability distributions rather than single points, and aims at identifying areas that show convergence across experiments. The results were corrected using the same statistical criteria as for the RS imaging data, i.e., using TFCE (43) and FWE-correction at *p* < 0.05.

#### Structural Covariance

Structural covariance was used to investigate the pattern of cortical gray-matter morphology across the whole brain by measuring the correlations of GMV, obtained through VBM, between different regions. This method assumes that such morphometric correlations carry some information about the structural or functional connectivity between the regions involved (13–15, 21). SC analysis was performed using the GMV estimates obtained from the VBM pipeline, as described above. Following preprocessing of the anatomical images, we first computed the volume of the seed region by integrating the (non-linear) modulated voxel-wise gray-matter probabilities of all voxels of the seed, which was then used as our covariate of interest for the group analysis. A whole-brain general linear model (GLM) analysis was applied using the GMV of the seed, along with the same additional covariates (of no interest) as for the RS-FC analysis. The results were corrected using the same statistical criteria as for the other connectivity modalities, i.e., using TFCE (43) and FWE-correction at *p* < 0.05.

#### Probabilistic Tractography

Probabilistic tractography was used to investigate white-matter anatomical connectivity from our seed region to the rest of the brain. The PT analysis was performed based on the same DWI as used for the TBSS analysis using the Diffusion Toolbox FDT implemented in FSL (16, 47). Fiber orientation distributions in each voxel were estimated according to Behrens et al. (48), i.e., using the BEDPOSTX crossing fiber model. Linear and subsequent non-linear deformation fields between each subject’s diffusion space and the MNI152 space as the location of the seeds and subsequent output were computed using the FLIRT and FNIRT tools, respectively. For PT, 100,000 samples were generated for each seed voxel and the number of probabilistic tracts reaching each location of a cortical gray matter. Importantly, we did not investigate the number of tracts reaching specific ROIs, but rather analyzed the number of tracts reaching each gray-matter voxel of the ICBM-152 template. The distance of each target (i.e., whole-brain gray matter) voxel from the seed voxel was computed using the ratio of the distance-corrected and non-corrected trace counts [cf. (49)]. This allowed us to address a limitation of structural connectivity profiles generated by PT, namely the fact that trace counts show a strong distance-dependent decay. That is, voxels close to the region of interest will inevitably feature higher connectivity values than even well-connected distant ones. These effects were adjusted by referencing each voxel’s trace count to the trace counts of all other gray-matter voxels in the same distance (with a 5-step, i.e., 2.5 mm, tolerance) along the fiber tracts [for a detailed description see Ref. (49)]. We thus replaced each trace count by a rank-based *z*-score indicating how likely streamlines passed a given voxel relative to the distribution of trace counts at that particular distance. The ensuing images were tested for consistency across subjects by using a second-level ANOVA. Results were corrected using the same statistical criteria as for the other connectivity modalities, i.e., using TFCE (43) and FWE-correction at *p* < 0.05.

#### Comparison of Connectivity Measures

The similarities and differences amongst all the different connectivity maps were compared and contrasted. The overlap between all the four thresholded connectivity maps (RS, MACM, SC, and PT) was computed using a minimum statistic conjunction (50), in order to identify *common connectivity* with the seed across the different modalities. This was done by computing the conjunction between the maps of the main effects for each of the modalities. An additional minimal conjunction analysis was also performed across the three modalities used to investigate gray-matter regions, namely, RS, MACM, and SC. Furthermore, we looked at *specifically present connectivity* for each of the modalities. *Specifically present connectivity* refers to regions that were connected with the seed in one modality but *not* in the other three [cf. (27)]. This was assessed by computing differences between the connectivity map of the first modality and those of the other three, respectively. Then a conjunction of these three different maps was performed. For example, the *specifically present connectivity* for MACM was assessed by computing the difference between the MACM map and the RS map in conjunction with the difference between the MACM map and the SC map and the difference between the MACM map and the PT map. Conversely, *specifically absent connectivity* was investigated by computing differences between one modality and the other three in order to identify regions that were present in the latter three modalities but not in the former. A conjunction of these different maps was then performed. For example, the *specifically absent connectivity* for MACM was assessed by computing the difference between the RS and MACM maps in conjunction with the difference between the SC and MACM maps and the difference between PT and MACM. All resulting maps were additionally thresholded with a cluster extent threshold of 100 voxels.

Finally, the resulting *common connectivity*, *specifically present connectivity* and *specifically absent connectivity* networks were functionally characterized based on the behavioral domain data from the BrainMap database as previously described for the seed region.

## Results

### Relationships Between TMT-MS Performance and Brain Structure: Whole-Brain VBM and TBSS Analyses

**Figure 2:**
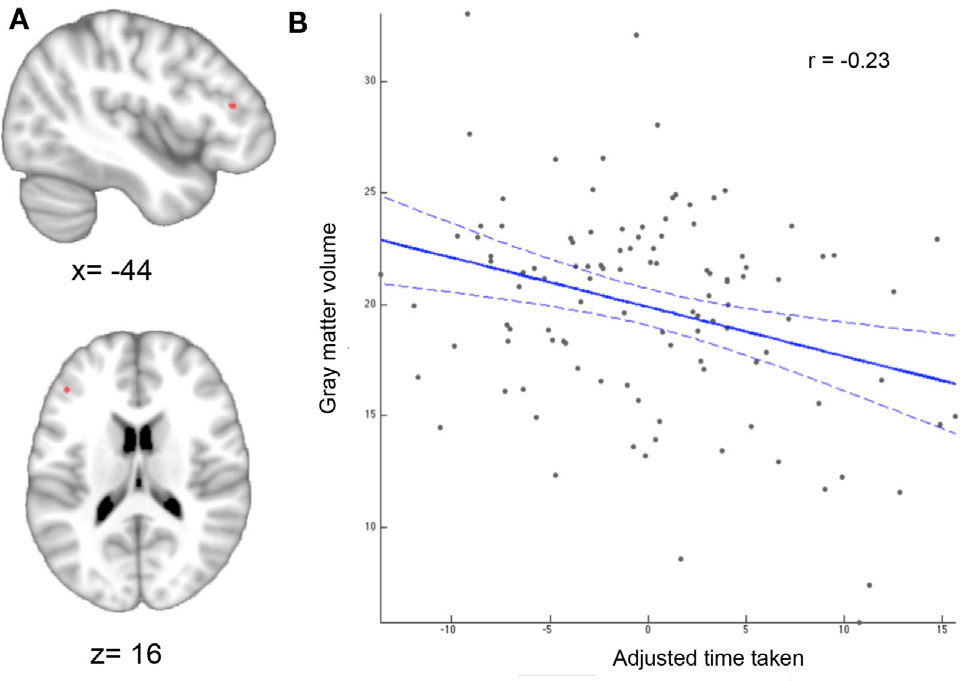
**Whole-brain VBM results**. **(A)** Region showing significant correlation between gray-matter volume and adjusted time taken. Statistical significance using non-parametric permutation inference was assessed at *p* < 0.05 [family-wise error (few) corrected for multiple comparisons]. **(B)** Correlation between motor speed and gray-matter volume. The better (lower) the performance score the higher the gray-matter volume.

The whole-brain VBM analysis revealed a significant negative correlation between the adjusted TMT-MS score and the GMV of a region in the lower bank of the left inferior frontal sulcus (IFS) Figure 2A). Since the TMT-MS score refers to task completion time, this negative correlation indicates that better performance was associated with higher GMV in this region (Figure 2B).

**Figure 3:**
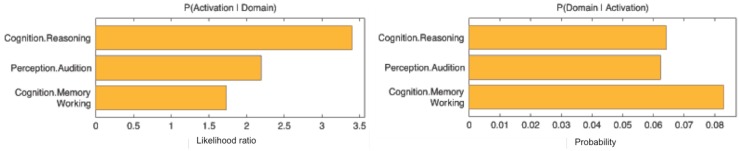
**Behavioral domains from the BrainMap database significantly associated with the seed, *p* **<** 0.05**.

The functional profile (based on the BrainMap database) of this region showed a significant association with cognition, specifically reasoning, at *p* < 0.05 (Figure 3).

The TBSS analysis of white-matter associations did not yield any significant results.

### Connectivity of the IFS

Whole-brain connectivity of the region showing a significant association with TMT-MS performance was mapped using RS-FC, MACM, SC, and PT. Both similarities and differences amongst all the different connectivity maps were observed.

#### Converging Connectivity

**Figure 4:**
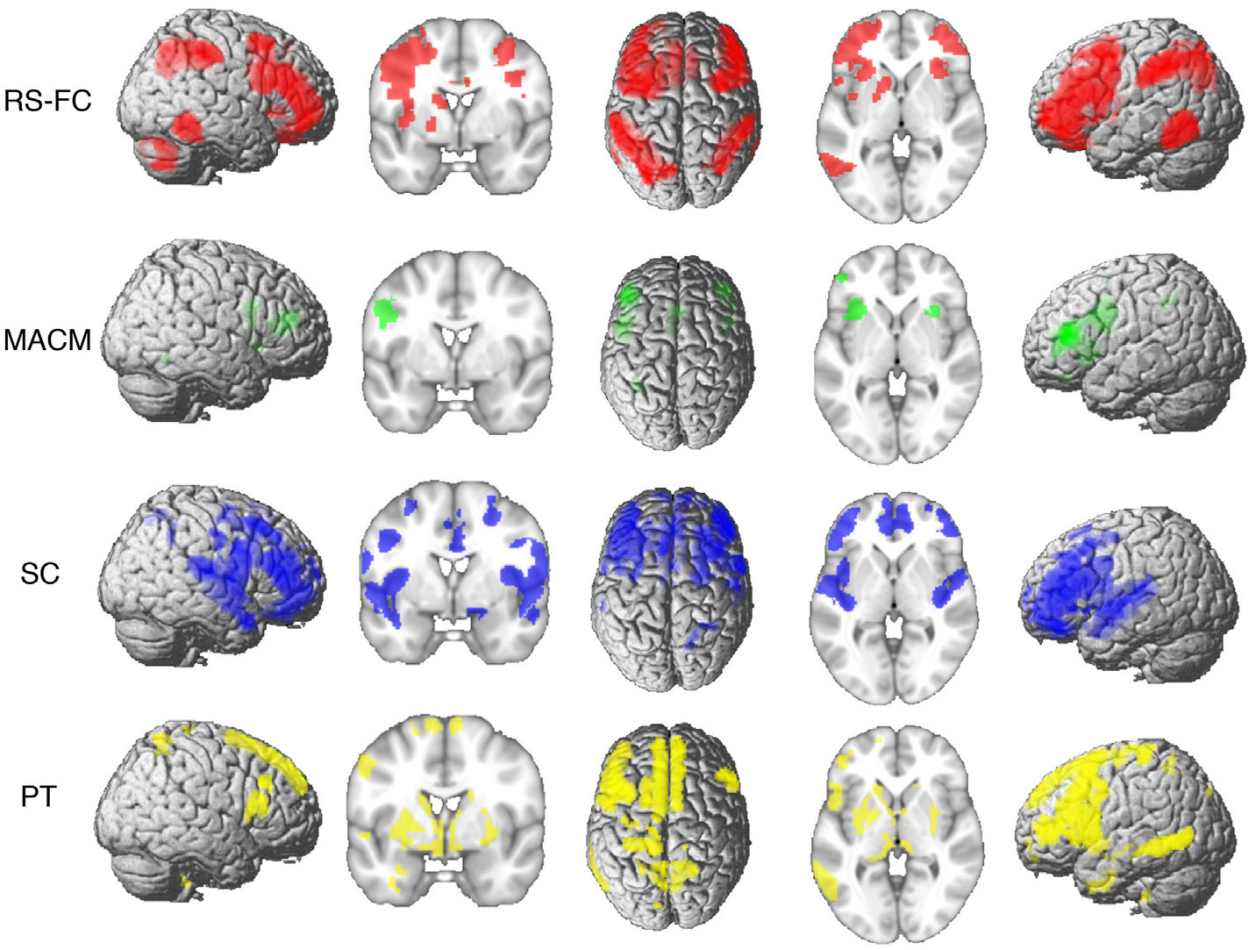
**Brain regions found to be significantly connected with the seed for each modality at *p* **<** 0.05, FWE corrected for multiple comparisons using threshold-free cluster enhancement (TFCE statistic)**.

**Figure 5:**
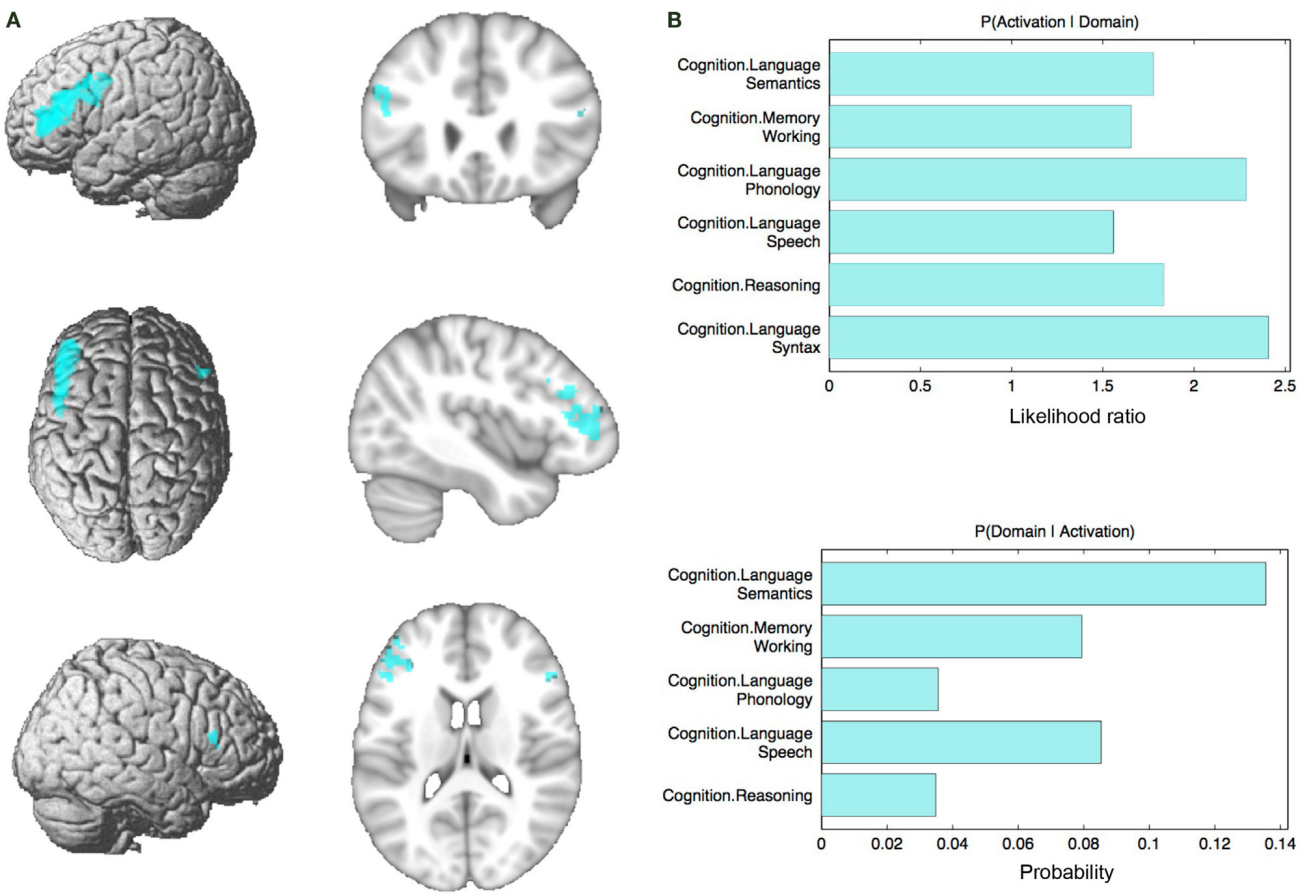
**Conjunction analysis and functional characterization of seed**. **(A)** Conjunction across RS-FC, MACM, SC, and PT. **(B)** Behavioral domains from the BrainMap database significantly associated with the commonly connected regions shown in **(A)** (FDR-corrected for multiple comparisons, *p* < 0.05).

**Figure 6:**
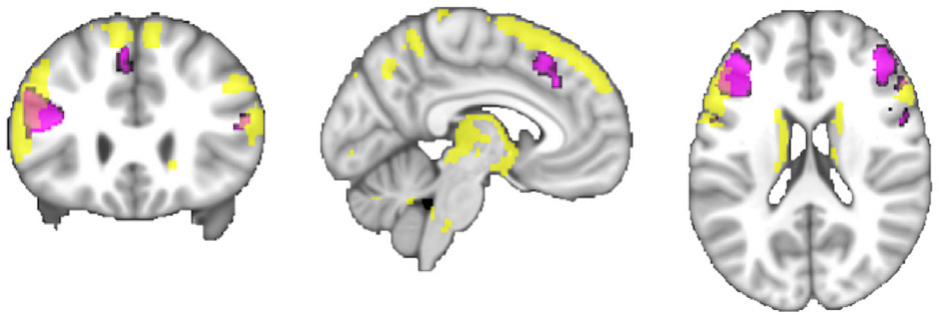
**A comparison of the conjunction across RS-FC, MACM, and SC (purple) with brain regions found to be significantly connected with the seed region when using PT (yellow) at *p* **<** 0.05, FWE corrected for multiple comparisons using threshold-free cluster enhancement (TFCE statistic)**.

Connectivity of the IFS seed, as revealed through RS-FC, MACM, SC, and PT analyses, included a number of distinct brain regions (Figure 4). Investigation of common regions interacting with the IFS across the different connectivity modalities (calculated through a minimum statistical conjunction analysis across the four thresholded connectivity maps) revealed convergence in the left inferior frontal gyrus (IFG) extending into the left IFS. An additional cluster was observed in the right Brodmann Area 45 (Figure 5A; Table 2). Functional characterization of this network found across all four connectivity approaches indicated an association with processes related to language, including semantics, phonology, and speech. Additionally, associations with working memory and reasoning were also revealed (Figure 5B). On the other hand, a conjunction across the modalities used to investigate gray-matter regions (RS-FC, MACM, and SC) resulted in a broader convergence, including clusters in the IFG bilaterally extending into the precentral gyrus, together with clusters in the middle cingulate cortex, middle orbital gyrus, and insula lobe of the left hemisphere (Figure 6).

**Table 2:** **Converging connectivity of the IFS seed**.

Region	*x*	*y*	*z*	Cytoarchitectonic assignment
**Cluster 1 (780 voxels)**				
L middle orbital gyrus	−46	46	−2	
**Cluster 2 (1,235 voxels)**				
R Inferior frontal gyrus (p. triangularis)	52	28	14	Area 45

**x*, *y*, and *z* coordinates refer to the peak voxel in MNI space. R, right; L, left*.

#### Specifically Present Connectivity for Each Modality

**Figure 7:**
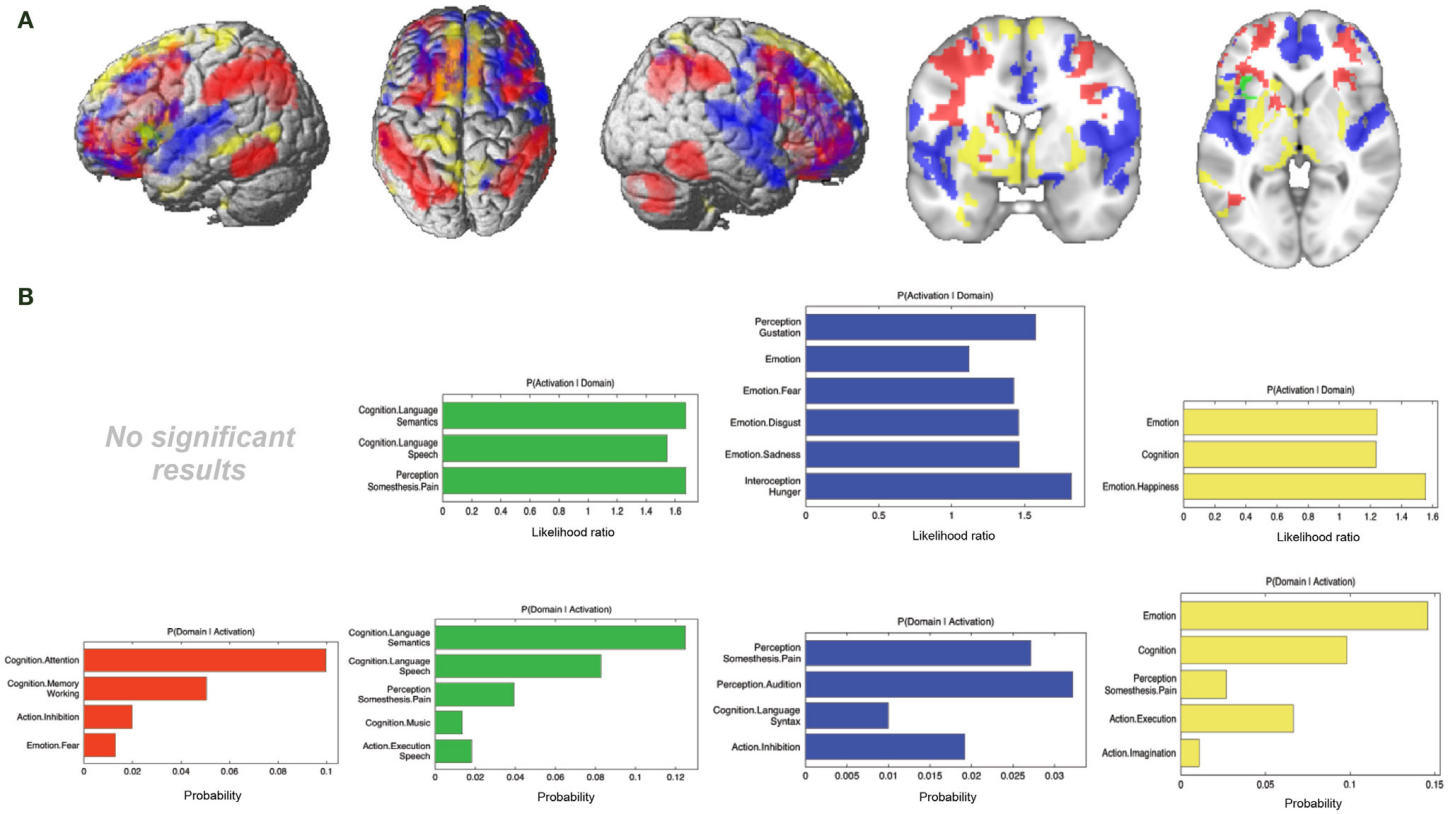
**Specific connectivity of seed and functional characterization**. **(A)** Specific connectivity for RS-FC (red), MACM (green), SC (blue), and PT (yellow). An additional cluster extent threshold of 100 voxels was applied. **(B)** Behavioral domains from the BrainMap database significantly associated with the specifically connected regions shown in **(A)** (FDR-corrected for multiple comparisons, *p* < 0.05).

In the next step, we looked at the connectivity effects that were present in one modality but not in the other three (Figure 7A; Table 3).

**Table 3:** **Specifically present connectivity of IFS seed**.

Region	*x*	*y*	*z*	Cytoarchitectonic assignment
**RS-FC**				
*Cluster 1 (5322 voxels)*				
L rectal gyrus	−4	24	−26	
*Cluster 2 (4183 voxels)*				
	−30	−72	20	
*Cluster 3 (3958 voxels)*				
	14	18	−28	
*Cluster 4 (2318 voxels)*				
	36	−64	24	
*Cluster 5 (1630 voxels)*				
R Cerebellum (Crus 2)	44	−66	−50	
*Cluster 6 (1357 voxels)*				
L inferior temporal gyrus	−52	−50	−26	
*Cluster 7 (817 voxels)*				
R inferior temporal gyrus	54	−50	−26	
**MACM**				
*Cluster 1 (279 voxels)*				
L insula lobe	−30	22	−10	
**SC**				
*Cluster 1 (26511 voxels)*				
R medial temporal pole	32	6	−33	
*Cluster 2 (7299 voxels)*				
	−39	3	−27	
*Cluster 3 (2577 voxels)*				
R superior frontal gyrus	21	33	30	
*Cluster 4 (1710 voxels)*				
L middle frontal gyrus	−40	51	10	
*Cluster 5 (875 voxels)*				
	−24	30	−23	
*Cluster 6 (525 voxels)*				
	28	−46	36	Area hIP1 (IPS)
*Cluster 7 (341 voxels)*				
L inferior frontal gyrus (p. Opercularis)	−57	15	7	Area 44
*Cluster 8 (229 voxels)*				
L SMA	−8	17	52	Area 6
*Cluster 9 (153 voxels)*				
L precentral gyrus	−33	−7	54	
*Cluster 10 (122 voxels)*				
L inferior frontal gyrus (p. Orbitalis)	−46	26	−5	
**PT**				
*Cluster 1 (919 voxels)*				
L superior medial gyrus	−8	54	28	
*Cluster 2 (748 voxels)*				
R superior medial gyrus	10	56	24	
*Cluster 3 (387 voxels)*				
L paracentral lobule	−10	−34	60	Area 4a
*Cluster 4 (308 voxels)*				
R precuneus	8	−66	40	Area 7A (SPL)
*Cluster 5 (234 voxels)*				
L inferior frontal gyrus (p. Orbitalis)	−48	22	−4	Area 45
*Cluster 6 (232 voxels)*				
L precuneus	−2	−72	36	Area 7P (SPL)
*Cluster 7 (179 voxels)*				
L middle temporal gyrus	−58	−28	−12	
*Cluster 8 (111 voxels)*				
	−4	−36	−48	
*Cluster 9 (107 voxels)*				
*L middle occipital gyrus*	−*52*	−*70*	−*2*	

**x*, *y*, and *z* coordinates refer to the peak voxel in MNI space. R, right; L, left*.

For RS-FC, we found specific connectivity between the seed region and bilaterally in the inferior parietal lobule, IFG (pars opercularis and pars triangularis), middle frontal gyrus, inferior temporal gyrus, middle orbital gyrus, and supramarginal gyrus. Additionally, areas in the right IFG (p. orbitalis), cerebellum, superior orbital gyrus, middle occipital gyrus, and angular gyrus were also revealed by RS-FC. Moreover, specific RS-FC connectivity was found in areas of the left superior parietal lobule (Figure 7A in red). When functionally characterized using the BrainMap meta-data (Figure 7B in red) the components of this network were found to be mainly associated with cognitive functions, including working memory, attention, and action inhibition. In addition, fear was also found to be associated with this network.

Connectivity exclusively found using MACM was only observed in one region in the left hemisphere, namely in the insula lobe and adjacent IFG (p. triangularis), in an area slightly more posterior position to that found in RS-FC (Figure 7A in green). This region was found to be mainly associated with language functions, namely semantics, speech, and speech execution. Moreover, functions such as pain perception and music were also found to be related (Figure 7B in green).

Connectivity specific to SC was observed in the bilateral superior medial gyrus, temporal pole, superior temporal gyrus, Heschl’s gyrus, rolandic operculum, supplementary motor area, superior and middle frontal gyri (more anterior to the effect found in RS-FC), IFG (p. orbitalis) (inferior to the area found in RS-FC on the right hemisphere) and middle orbital gyrus (bilaterally more anterior to the RS-FC effect). In the right hemisphere, specifically present SC connectivity included areas in the anterior cingulate cortex, insula lobe, middle temporal gyrus, supramarginal gyrus (more inferior to the area found in RS-FC), medial temporal pole, superior and inferior parietal lobules (the latter being more inferior to the area found in RS-FC), and superior orbital gyrus (more anterior to RS-FC specific connectivity in the same region). Additional connectivity was also observed in the left rectal gyrus, and left precentral gyrus (Figure 7A in blue). This network was found to be mainly functionally associated with functions related to emotion (fear, disgust, and sadness) and perception (audition and pain) (Figure 7B in blue).

The network specifically present for PT was found to be mainly functionally associated with functions related to emotion and pain. Additionally, functions such as action execution and action imagination were also found to be related (Figures 7A,B in yellow).

#### Specifically Absent Connectivity for Each Modality

**Figure 8:**
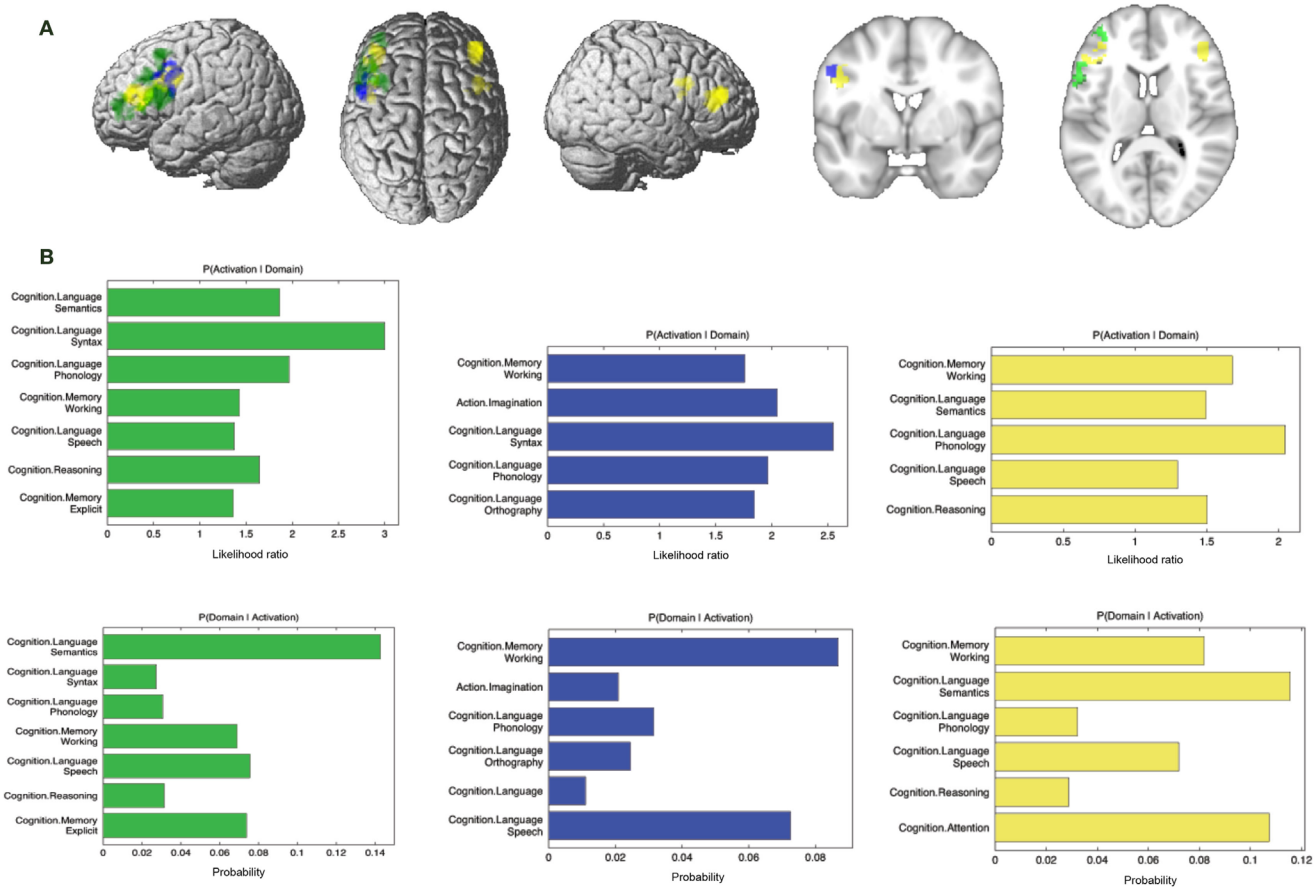
**Specifically missing connectivity of seed and functional characterization**. **(A)** Specifically missing connectivity for MACM (green), SC (blue), and PT (yellow). An additional cluster extent threshold of 100 voxels was applied. **(B)** Behavioral domains from the BrainMap database significantly associated with the specifically missing regions shown in **(A)** (FDR-corrected for multiple comparisons, *p* < 0.05).

Additionally, we looked at connectivity that was specifically absent in each modality, i.e., regions for which connectivity was absent in a particular modality but was observed in the other three (Figure 8A; Table 4). No regions were found to be specifically absent for the RS-FC modality. By contrast, for MACM we found specifically absent connectivity with areas of the left middle and inferior frontal gyri (p. triangularis) (Figure 8A in green). These regions were found to be functionally associated with cognitive functions, namely working and explicit memory but also with phonology, semantics, and syntax (Figure 8B in green).

**Table 4:** **Specifically absent connectivity of IFS seed**.

Region	*x*	*y*	*z*	Cytoarchitectonic assignment
**MACM**				
*Cluster 1 (735 voxels)*				
L inferior frontal gyrus (p. triangularis)	−42	40	−2	
L inferior frontal gyrus (p. triangularis)	−50	38	6	
L inferior frontal gyrus (p. triangularis)	−52	20	30	Area 45
*Cluster 2 (166 voxels)*				
L middle frontal gyrus	−44	12	38	Area 44
**SC**				
*Cluster 1 (205 voxels)*				
L precentral gyrus	−50	4	16	
**PT**				
*Cluster 1 (629 voxels)*				
L inferior frontal gyrus (p. triangularis)	−42	32	6	
*Cluster 2 (339 voxels)*				
R inferior frontal gyrus (p. triangularis)	46	34	6	Area 45
*Cluster 3 (119 voxels)*				
R precentral gyrus	54	6	18	Area 44

**x*, *y*, and *z* coordinates refer to the peak voxel in MNI space. R, right; L, left*.

Conversely, for SC specifically absent connectivity was found for an area in the left precentral gyrus (Figure 8A in blue; Table 4). This region was in turn found to be mainly functionally associated with language-related functions (phonology, semantics, speech, and syntax) together with working memory (Figure 8B in blue).

Connectivity specifically absent for PT was also found to be functionally associated with language-related functions (phonology, semantics, and speech) together with working memory, reasoning, and attention (Figures 8A,B in yellow).

## Discussion

The aim of this study was to employ a multi-modal approach to investigate the regions and associated networks related to upper extremity motor function, as quantified by the TMT-MS. In a first step, we therefore correlated local GMV with performance in motor speed. This analysis revealed a significant correlation between TMT-MS performance and GMV in a small region in the IFS, which was functionally characterized as being involved in cognitive tasks. In turn, the TBSS analysis of local WM associations yielded no significant result. We then further investigated the connectivity of the left IFS seed using a multi-modal approach. Functional interactions with other gray-matter regions and white-matter structural connections were assessed using RS-FC, MACM, SC, and PT approaches. The networks that emerged revealed both similarities and differences between the different modalities. A conjunction analysis between the four connectivity approaches was used to delineate a core network. Further analyses were used to investigate connectivity patterns specific to each of the modalities.

### Relationships Between TMT-MS Performance and Brain Structure

In this study, we found TMT-MS performance to be specifically related to the local brain volume of a region in the lower bank of the left IFS. That is, across subjects better performance (lower completion time) was associated with higher GMV in this cluster. The left IFG, including IFS, has been formerly described as part of a multiple-demand system responsible for multiple kinds of cognitive demand, in which goals are achieved by assembling a series of sub-tasks, each separately defined and solved (51). An objective definition of this “multi-demand network” has recently been proposed by Müller et al. (52) based on a conjunction across three large-scale neuroimaging meta-analyses to identify regions consistently involved in sustained attention (53), working memory (54), and inhibitory control (55). Importantly, the IFS location identified in the current study was found to be part of this multi-demand network, indicating that TMT-MS performance is related to brain structure in a region involved in executive rather than motor functions. This association between certain aspects of motor performance and cognitive or executive functions has already been suggested in earlier studies (56, 57).

At first glance, these results contradict the intention of the TMT-MS to measure motor speed, and to serve as a baseline measure for higher, executive aspects of the test (2). However, one may argue that since subjects are given specific instructions to follow a dotted line while making sure that the line drawn touches every circle along the path, the accurate completion of this task should in fact draw heavily on executive control processes. It may hence not surprise that performance in a task requiring a relatively high degree of executive motor control and attention is related to a structure that is part of the multi-demand network involved in executive functions (51). In turn, there was no significant association between performance and GMV in cortical or subcortical motor structures as may have been expected. In this context, it must be noted that adequate hand motor abilities are a necessary prerequisite for performing the TMT-MS test successfully; i.e., subjects have to be able to use their hand to draw the required lines. Hence, the reliance of TMT-MS completion on an intact cortical and subcortical motor system is obvious. What we found, however, is that performance (i.e., the speed at which the task is completed) may seem to primarily depend on executive rather than more basic motor control processes. Does this contradict the assumption that the TMT-MS test is a baseline measure of motor speed? Not necessarily, but rather, given our findings, we would argue that motor speed in a more naturalistic setting should be more strongly associated with executive rather than primary motor function.

In congruence with the present results, previous studies have linked longer reaction times and motor slowing with sustained attention (58). However, lesion studies have associated slowing in motor processes with lesions in the right lateral frontal lobe (59, 60). Consequently, these results contrast with the findings of the present study. Additionally, the present results differ from those obtained using tasks that are commonly employed to investigate changes to the motor system following stroke; for instance, in functional neuroimaging studies using fist opening/closure paradigms (61, 62). Here, activation and interactions of the primary motor cortex as well as the lateral and medial pre-motor cortices are of essential importance. Similar regions were found in another functional neuroimaging study which used a finger tapping paradigm and focused on healthy subjects (63). In turn, activations involving the inferior frontal cortex and other regions of the executive, multi-demand network are not prominently seen. This implicates a potentially important distinction between neuroimaging assessments of stroke patients, in which more fundamental aspects of motor performance are usually tested, and paper-and-pencil tests that apparently, even when aimed at testing basic motor speed, are more reflective of executive motor control. In summary, we would thus argue that the distinction between motor and “higher cognitive” tasks, which seems rather prevalent in (neuroimaging) stroke research, may be slightly misleading, as executive motor control functions may play a major role in the everyday impairments following stroke.

#### Core Network

Notably, all three FC approaches (RS-FC, MACM, and SC), together with locations revealed as structurally connected by PT, converged on a network comprising of the left inferior gyrus extending into the left IFS and an additional cluster in the right Brodmann Area 45. In combination with the observation of a fairly restrictive region associated with TMT-MS performance, these results suggest a core network of mostly regional connectivity that is in line with the current view on the role of the inferior frontal cortex in executive functioning (51).

Additionally, the right IFG, bilateral adjacent pre-motor cortices, and anterior insula were additionally found to converge when looking only at the FC approaches, namely, RS-FC, MACM, and SC (but not PT). Similar as the IFS seed, most of these clusters overlap with regions previously described to be part of the multiple-demand network (51, 52). In particular, the bilateral IFG, and left anterior insula as well as the MCC were the regions that overlapped with the multiple-demand network. Thus, we here show that, across different (functional) connectivity approaches the IFS shows robust interactions with regions associated with multiple cognitive demands. This is additionally supported by the functional characterization of the network robustly connected with the IFS across the different FC approaches, which show an association with multiple cognitive tasks. These observations thus continue to emphasize the important role of cognitive functions in the TMT-MS and thus suggest that this test might be tapping into executive rather than primary motor function.

### Convergence and Differences Between Connectivity Measures

#### Convergence Among Modalities

Functional interactions can be probed by using different approaches, each having their own methodological features, and potentially also different biases even though the same statistical analyses and thresholds were used for each of the modalities. The use of the different modalities in the current study provided an opportunity to systematically compare all the different approaches. Despite the conceptual differences between the different modalities, a common network was revealed. When comparing the modalities RS-FC, MACM, and SC networks through a minimum statistic conjunction analysis, all three approaches converged on a core network that included adjacent parts of left IFG, its right-hemispheric homolog, right precentral gyrus, left middle cingulate cortex, middle orbital gyrus, and insular cortex. These results are in line with previous studies that used different seeds and therefore different networks, and also showed convergence between RS and MACM (18–20), between RS and SC (21, 22, 28), between RS and fiber tracking (23–26), and between RS, MACM, and SC (27, 64). As a result, it can be suggested that future studies could benefit from a multi-modal approach and the consequent use and interpretation of the convergent network rather than focusing on a unimodal approach.

Furthermore, our resulting similarity between the SC and PT networks and the networks obtained from the other two modalities supports the idea that FC can be used to reflect structural connectivity and that SC of GMV can reflect functional networks in the brain (21, 22, 27). Consequently, our results together with previous findings provide evidence for the fact that SC is functional in nature.

#### Differences Among Modalities

Despite the convergence observed across all approaches, divergent connectivity patterns were also found when looking at contrasts of the different modalities. This is not surprising, given that the approaches use different data and methods in order to determine connectivity between a seed region and the rest of the brain. Previous studies have similarly reported striking differences between RS-FC and MACM connectivity approaches (20, 27). Clos et al. (27) and Jakobs et al. (20) have already argued that the differences that result from these two approaches may be the result of the conceptual differences between the methods. While RS-FC is based on correlation of fMRI time-series measured in the absence of an external stimulus (5, 65), MACM delineates networks that are conjointly recruited by a broad range of tasks (3). That is, RS and MACM derive FC from different mental states, in the absence and presence of a task, respectively. As a result, spontaneous networks related to self-initiated behavior and thought processes that can be captured in the task-free state may be largely missed in MACM analyses (3).

In particular, RS-FC of our seed was specifically found in a number of regions that have been predominantly associated with executive functions, such as working memory, attention, action inhibition, and spatial cognition. Importantly, there were no regions that were present in SC, PT, and MACM, but absent in RS-FC as revealed by the specifically absent RS-FC. This indicates that RS-FC captures the broadest network. By contrast, specific connectivity observed for MACM was found to be mainly associated with language-related functions such as semantics and speech. In turn, specifically absent regions in MACM were found to be mainly associated with cognitive functions such as working memory and explicit memory as well as language-related functions. As already mentioned above, these diverging patterns, with RS-FC capturing a broader network than MACM is possibly due to the conceptual differences. Moreover, these two approaches also differ in the subject groups assessed. While a group of 109 subjects were recruited for the RS-FC analysis, the MACM analysis relied on a large amount of published neuroimaging studies from the BrainMap database (10), with the selection criteria being activation of our identified seed region. Thus, it is possible that this difference in subject groups may have also contributed to the difference in results obtained.

In contrast to the FC approaches mentioned above, specific SC connectivity was observed in regions found to be mainly associated with functions related to emotion (fear, disgust, and sadness) and perception (pain, gustation, audition, hunger, and somesthesis). Additional functions observed included action inhibition and cognition. On the other hand, functional characterization of areas that were found to be specifically absent for SC connectivity revealed an association with functions related to cognition and language such as working memory, phonology, orthography, syntax, and speech. Given these results, it can be noted that the specific SC network showed a prominent association with perception and emotional processing. The strong association with emotional processing in SC is particularly interesting since the functional characterization of the seed region and the conjunction network did not indicate such an involvement. Moreover, while the specific RS-FC network revealed regions that were predominately related to cognition and the MACM network revealed regions that were predominantly related to language, the SC network found such regions to be specifically missing. These differences may be largely due to the conceptual differences between the FC modalities described above and SC. The exact biological basis of SC is still rather unclear (27), but it has been hypothesized that SC networks arise from synchronized maturational change that could be mediated by axonal connections forming and reforming over the course of development (66). Therefore, early and reciprocal axonal connectivity between regions is expected to have a mutually trophic effect on regional growth in an individual brain leading to covariance of regional volumes across subjects (14). That is, the correlation of anatomical structure between regions is the result of similarities in maturational trajectories (14). The specific connectivity pattern of the SC modality may thus be reflecting synchronized developmental patterns within a network of regions associated with perception and emotional processing. This could thus be the reason for particular regions to be present in the SC network and not in the MACM and RS-FC networks since the latter two modalities are more likely to highlight regions that are related to certain functions rather than long-term anatomical interactions. Additionally, SC is also likely to include other influences such as common genetic factors, developmental brain symmetry, neuromodulator distributions, and vascular territories (14, 15), which contribute to its more widespread distribution.

In congruence with the specific SC network, the PT network also showed a prominent association with perception and emotional processing while functional characterization of areas that were found to be specifically absent for PT connectivity revealed an association with functions related to cognition and language. These results further imply that the regions that were specifically associated with SC may reflect dominant long-term synchronized maturational patterns. However, despite the differences observed, it should be noted that the core network showed that the resulting SC network (also) revealed functional relations despite the fact that it was defined by anatomical covariance. SC may hence be regarded as a measure potentially bridging between structural and functional connectivity aspects. However, when comparing the PT to the other three networks, contrasting regions can be observed. This could be due to biases related to the use of conventional diffusion tensors. Such tensors can only capture the principal diffusion direction, and thus makes them prone to errors induced by crossing fibers (67). As a result, this could have limited the possible resulting convergence amongst the four modalities.

## Conclusion

In summary, the present results demonstrate a significant correlation between TMT-MS performance and GMV in the lower bank of the IFS, which was functionally characterized as being involved in cognitive tasks. Additionally, all connectivity approaches used (RS-FC, MACM, SC, and PT) converged on a network comprising of regions that overlap with the multiple-demand network. Results therefore indicate that performance (i.e., the speed at which the task is completed) may primarily depend on executive function, thus suggesting that motor speed in a more naturalistic setting should be more strongly associated with executive rather than primary motor function. Moreover, the common connectivity resulting from the different modalities used verifies that common networks can be revealed across highly divergent methods.

## Conflict of Interest Statement

The authors declare that the research was conducted in the absence of any commercial or financial relationships that could be construed as a potential conflict of interest.

## Footnotes

^1^http://fcon_1000.projects.nitrc.org/indi/enhanced

^2^www.fmrib.ox.ac.uk/fsl

^3^http://www.brainmap.org

^4^http://www.fil.ion.ucl.ac.uk/spm/software/spm8/

^5^http://www.brainmap.org
